# Prognostic Factors for Vulvar Cancer Undergoing Primary Surgery: Case Series from a Single Tertiary Cancer Center

**DOI:** 10.1007/s13193-024-02134-2

**Published:** 2024-12-06

**Authors:** T. S. Shylasree, Ushashree Das, Neha Kumar, Lavanya Naidu, Kedar Deodhar, Supriya Chopra, Pabashi Poddar, Amita Maheshwari

**Affiliations:** 1 Department of Gynecological Oncology, Tata Memorial Hospital, Homi Bhabha National Institute, Room No. 1211, Homi Bhabha Block, Dr Ernest Borges Marg, Parel, Mumbai, 400012 Maharashtra India; 2 Department of Gynecological Oncology, Royal Aberdeen Infirmary, Foresthill Estate, Aberdeen, AB25 2ZN Scotland; 3 Department of Gynecological Oncology, Bagchi Sri Shankara Cancer Centre and Research Institute, Bhubaneswar, 752054 India; 4 Department of Gynecological Oncology, Amrita Institute of Medical Sciences and Research Centre, Faridabad, 121002 India

**Keywords:** Vulvar cancer, Tumor-free margin, Groin node metastases, Lympho-vascular space invasion

## Abstract

To evaluate clinical outcomes in women undergoing primary surgery for vulvar squamous cell carcinoma) (SCC) with an aim to identify surgico-pathological risk factors associated with recurrence and survival. Retrospective cohort analysis was carried out between January 2011 and December 2018 for patients with vulvar SCC who underwent primary surgery. The Kaplan–Meier method was used for the estimation of the probability of disease-free survival (DFS) and overall survival (OS). Univariate and multivariate analyses based on the Cox proportional hazards model were performed to identify factors associated with DFS and OS. A *p*-value ≤ 0.05 in a two-tailed test was considered statistically significant. The study population included 81 patients; the median follow-up time for the entire cohort was 41 months. Recurrence was noted in 27 cases (33.3%), and the median time to recurrence was 36.14 months. The median overall survival (OS) was 40.8 months, and disease-free survival (DFS) was 36 months. On univariate analysis, depth of invasion (DOI), close margin, presence of lympho-vascular space invasion (LVSI), perineural invasion (PNI), groin metastases, and not receiving adjuvant therapy were significantly associated with increased recurrence rates. Deeper stromal invasion, presence of LVSI, groin node metastases, and recurrent disease were associated with poor OS on univariate analysis. On multivariate analysis, DOI, tumor-free margin (TFM), and PNI were significantly associated with DFS, and a previous history of recurrence was associated with OS. Vulvar cancers are relatively rare tumors with several local tumor factors such as TFM, DOI, LVSI, and lymph node status which may help determine oncological outcomes. Larger studies will definitely help establish more evidence.

## Introduction

The rarity of vulvar cancer (VC) is well known; it constitutes less than 5% of all gynecologic malignancies [1]. The Globocan 2020 data reported 3447 new cases in India, with a five-year prevalence of 8928 [2]. Surgical excision, with or without groin node dissection, is the treatment of choice in early-stage VC.

The revision to vulvar cancer staging by the FIGO Committee for Gynecologic Oncology in 2009 made substantial changes to stage assignment, including the following: (1) disease involvement of the lower urethra, vagina, and anus was assigned to Stage II; (2) all nonmetastatic tumors were collectively assigned to Stage I; and (3) the number and extent of lymph node involvement was extensively substaged within Stage [3].

Local recurrences are common in early-stage VC, with a reported incidence close to 37–40% [4, 5]. Adjuvant treatment, mainly radiotherapy, is based on the presence of adverse histopathological parameters. Identification of these prognostic factors is crucial for reducing recurrences and tailoring surgical treatment. Stage and lymph node status are the most accepted risk factors for recurrence and survival [1, 4]. Other risk factors known for locoregional recurrences include lesion size, depth of invasion (DOI), close surgical margins, lympho-vascular space invasion (LVSI), and perineural invasion (PNI). With the limited availability of large patient cohort studies, it is challenging to demonstrate the significance of the above factors. It is imperative to comprehensively collate the available literature for conclusive results.

In this study, we evaluated clinical outcomes in women undergoing primary surgery for operable vulvar squamous cell carcinoma (SCC) to identify the surgico-pathological risk factors associated with recurrence and survival.

## Materials and Methods

This retrospective cohort analysis included patients with operable vulvar squamous cell carcinoma (SCC) (FIGO I-IIIC [International Federation of Gynecology and Obstetrics]) who underwent primary surgery at a tertiary cancer care center between January 2011 and December 2018. Patients with extensive locoregional disease deemed unresectable, those primarily treated with radiotherapy, or those who received salvage surgery after neoadjuvant radiotherapy or chemotherapy were excluded. Patients with lateralized lesions underwent ipsilateral sentinel lymph node dissection or groin node dissection. Patients with anterior or posterior central vulvar lesions underwent bilateral sentinel lymph node dissection or groin node dissection. All patients undergoing groin node dissection had a groin wound drain, which was removed when drainage was minimal. Flap-based reconstruction was used to treat major perineal defects.

All information was retrieved from electronic medical records after approval by the Institutional Review Board’s Ethics Committee (IEC No.: 3652). The 2009 FIGO staging system for vulvar cancers was used. Electronic medical records provided individual patient information. Clinico-demographic data, treatment details, survival outcomes, and follow-up data were gathered. The following clinicopathological data were extracted: age, tumor stage, grade, size, DOI, LVSI, PNI, positive or close surgical margin, and lymph node status along with the number of positive nodes. All patients underwent radical local excision with or without groin node dissection. Adjuvant radiotherapy to the perineum was administered for a close margin (< 5 mm) at the primary tumor site, and radiotherapy to the bilateral groins and pelvis, with or without chemotherapy, was administered for more than one groin node metastasis. All patients were followed up regularly with clinical evaluations every 4 months for the first 2 years, followed by every 6 months for the next 3 years. Recurrence in this study included local, locoregional, and distant recurrence diagnosed clinically or by imaging. Histopathologic diagnosis of recurrence was not necessary with unequivocal imaging or clinical findings. Age, tumor size, tumor-free margins (TFM—the closest distance from the invasive tumor to the resection margin in paraffin section), DOI, lymph node status, and stage were identified as risk factors for assessing recurrence patterns.

### Statistical Analysis

Statistical analyses were performed using SPSS (Statistical Package for the Social Sciences) version 25.0 (IBM Corp.). Disease-free survival (DFS) was defined as the time from surgery to recurrence, death from any cause, or last follow-up. Overall survival (OS) was defined as the time from surgery to death from any cause or last follow-up. The Kaplan–Meier method was used to estimate DFS and OS probabilities. Univariate and multivariate analyses, based on the Cox proportional hazards model, were performed to identify factors associated with DFS and OS. A *p*-value ≤ 0.05 in a two-tailed test was considered statistically significant.

## Results

The study population included 81 patients treated primarily with surgery between January 2011 and December 2018. The median follow-up time for the entire cohort was 41 months (range: 2–106 months). Baseline characteristics and surgico-pathological features of the study population are described in Table 1.

**Table 1:** Baseline characteristics and treatment details of study population

Variable	Number (*n*) (%)
Median age in years (range)	55 (27–89)
Lesion location	
Labia majora	46 (56.8%)
Labia minora	14 (17.3%)
Clitoris	9 (11.1%)
Perineum	3 (3.7%)
Multifocal	9 (9.9%)
FIGO stage 2009 [5]	
IA	13 (16%)
IB	43 (53.1%)
II	5 (6.2%)
III	19 (23.5%)
Staging information NA	1 (1.2%)
Grade	
I	16 (19.8%)
II	47 (58%)
III	13 (16%)
Not available	5 (6.2%)
Tumor size	
Not known	8 (9.9%)
≤ 2 cm	18 (22.2%)
> 2 cm	55 (67.9%)
Depth of invasion (DOI)	
≤ 8 mm	51 (63.0%)
> 8 mm	7 (8.6%)
Information not available	23 (28.4%)
Lympho-vascular space invasion (LVSI)	
Present	5 (6.2%)
Absent	62 (76.5%)
Information not available	14 (17.3%)
Perineural invasion (PNI)	
Present	5 (6.2%)
Absent	60 (74.1%)
Information not available	16 (19.8%)
Pathological tumor-free margin (TFM)	
≥ 1 cm	54 (66.7%)
< 1 cm	23 (28.4%)
Information not available	4 (4.9%)
Groin node metastases	
Not addressed	18 (22.2%)
Present	19 (23.5%)
Absent	44 (54.3%)
Surgery for primary tumor	
Radical local excision	50 (61.7%)
Total radical vulvectomy	31 (38.3%)
Type of groin surgery	
Not addressed	18 (22.2%)
Sentinel node dissection	12 (14.8%)
Bilateral complete groin node dissection	43 (53.1%)
Unilateral complete groin node dissection	3 (3.7%)
Groin node debulking/selective sampling	5 (6.2%)
Staging information NA	1 (1.2%)
Adjuvant treatment	
No adjuvant treatment	62 (76.5%)
Radiation	13 (16%)
Chemotherapy + radiation	25 (6.2%)
Chemotherapy	1 (1.2%)
Status at last follow-up	
Dead	14 (17.3%)
Alive with disease	7 (8.6%)
Alive without disease	54 (66.7%)
No information available	6 (7.4%)

*FIGO* International Federation of Obstetrics and Gynecology, *DOI* depth of invasion, *LVSI* lympho-vascular space invasion, *PNI* perineural invasion, *TFM* pathological tumor-free margin, *NA* not available

### Treatment Details (Table 1)

The primary tumor was excised with radical local excision in 61.7% of cases when a satisfactory tumor-free margin was feasible, while 38.3% of cases underwent radical vulvectomy because of the size or location of the primary tumor (Table 3). A tumor-free margin was obtained in 66.7% of cases (54 cases). Bilateral groin node dissection (GND) was performed in 53.1% of cases, while sentinel lymph node dissection using the dual-dye method (methylene blue and indocyanine green) was performed in 14.8% of cases. Unilateral groin node dissection was performed in only 3 cases (3.7%), while 5 cases (6.2%) underwent only groin node debulking because of the presence of metastatic nodes. Nodal dissection was not performed in 22.2% of cases. Adjuvant treatment was given in 39 (23.4%) cases; 68.4% of these patients received only radiation, 26.3% received chemoradiation (CTRT), and one patient received only chemotherapy (CT).

Postoperative wound dehiscence (local or groin) was observed in 18 cases (22.2%), and six cases (7.4%) developed chronic lymphedema. One patient developed a chronic lymphocyst, which was managed conservatively. One patient developed chronic neuropathic pain in the lower limb, requiring long-term analgesics.

### Patterns of Recurrence

**Fig. 1:**
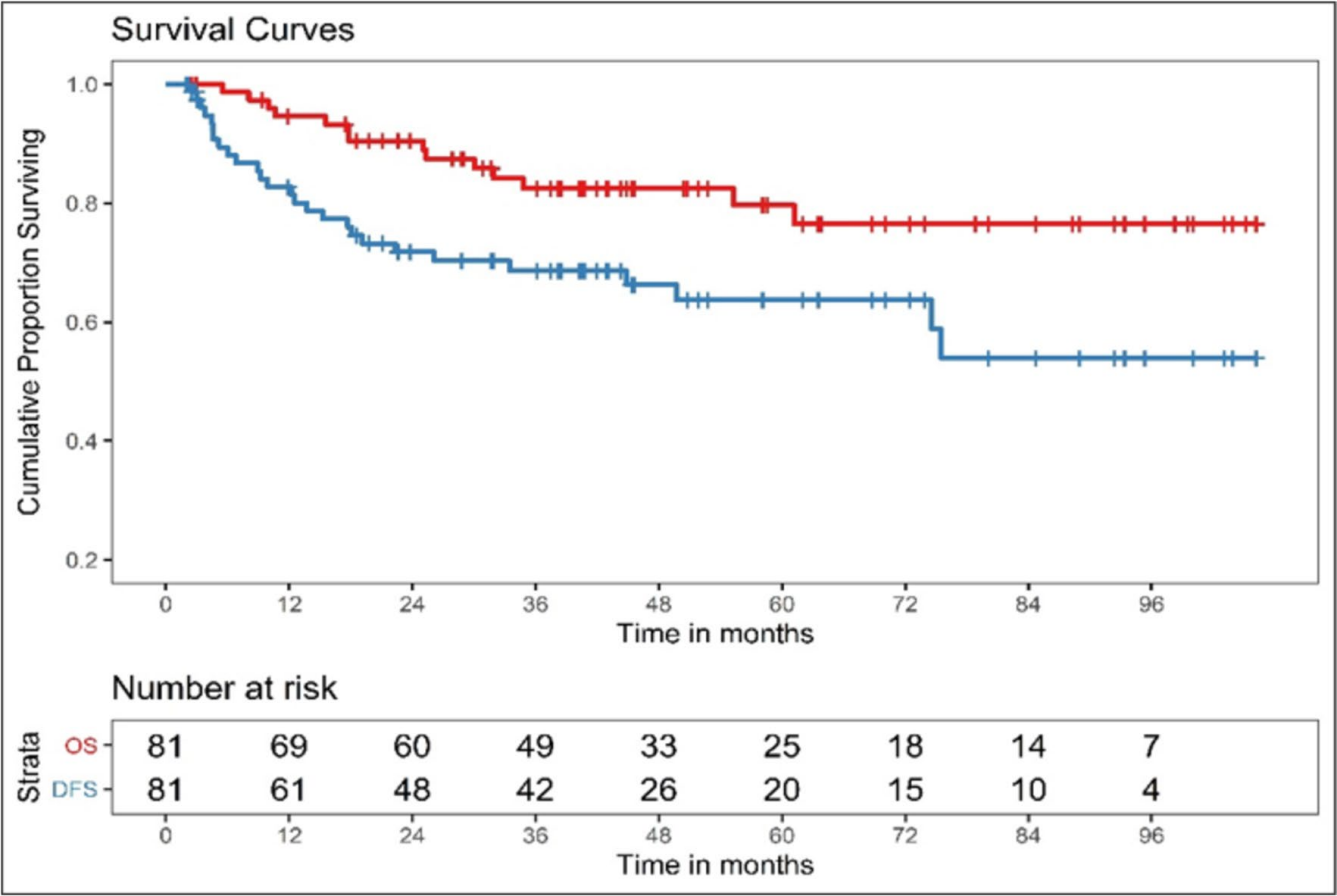
Kaplan-Meir curves for DFS and OS

Recurrence was noted in 27 cases (33.3%), and the median time to recurrence was 36.14 months (range: 2–106 months). Local recurrence was the most common type of recurrence (48.1%), and 3 cases (11.1%) developed groin node recurrences. Six cases (22.2%) had both local and groin node recurrence, and 5 cases (18.5%) developed distant recurrences (including pelvic lymph node recurrence). Nearly half of the recurrences (13 cases—48%) were treated with curative intent using surgery/radiotherapy, with or without chemotherapy, whereas 14 cases (52%) were given palliative care/palliative chemotherapy or palliative radiotherapy (RT) due to a high disease burden or poor patient performance status. The median overall survival (OS) was 40.8 months, and the median disease-free survival (DFS) was 36 months. Kaplan–Meier estimates for 3-year OS were 82.5% (95% CI: 71.13%–89.71%), and for 3-year DFS were 68.64% (95% CI: 56.61%–77.97%), as shown in Fig. 1.

On univariate analysis, DOI, close margin, presence of LVSI, PNI, groin metastases, and not receiving adjuvant therapy were significantly associated with increased recurrence rate. Deeper stromal invasion, presence of LVSI, groin node metastases, and recurrent disease were associated with a poor OS on univariate analysis. On multivariate analysis, DOI, TFM, and PNI were significantly associated with DFS, and previous history of recurrence was associated with OS (Tables 2 and 3).

**Table 2:** Univariate and multivariate analysis for DFS

	Univariate analysis for DFS HR (95% CI); *p*-value	Multivariate analysis for DFS HR (95% CI); *p*-value
Age	1.289 (0.587–2.831); 0.526	
Tumor size (≤ 2 cm vs. > 2 cm)	1.6 (0.6–4.5); 0.341	
DOI (≤ 8 mm, > 8 mm)	1.1 (1.0–1.3); 0.030	36.853 (2.109–644.13); 0.013
TFM (≥ 1 cm, < 1 cm)	3.8 (1.7–8.5); 0.001	4.0 (1.4–11.2); 0.008
LVSI (present, absent)	2.7 (0.8–9.1); 0.113	
PNI (present, absent)	4.7 (1.5–14.5); 0.007	6.704 (1.260–35.679); 0.026
Groin node metastases (present, absent)	11.5 (3.7–35.8); 0.000	5.503 (.661–45.847); 0.115
Adjuvant treatment (yes, no)	3.0 (1.4–6.6); 0.005	0.694 (0.180–2.680); 0.597

*DFS* disease-free survival, *DOI* depth of invasion, *LVSI* lympho-vascular space invasion, *PNI* perineural invasion, *TFM* pathological tumor-free margin, *NA* not available

**Table 3:** Univariate and multivariate analysis for OS

	Univariate analysis for OS HR (95% CI); *p*-value	Multivariate analysis for OS HR (95% CI); *p*-value
Age	1.681 (0.580–4.868); 0.338	
Tumor size (≤ 2 cm vs > 2 cm)	4.6 (0.6–36.1); 0.143	
DOI (≤ 8 mm, > 8 mm)	1.3 (1.1–1.5); 0.000	2.312 (0.211–25.323) 0.493
TFM (≥ 1 cm, < 1 cm)	1.9 (0.6–6.1); 0.267	
LVSI (present, absent)	8.3 (2.2–31.6); 0.002	3.572 (0.458–27.853); 0.947
PNI (present, absent)	3.3 (0.7–15.6); 0.125	
Groin node metastases (present, absent)	7.4 (1.8–29.8); 0.005	0.094 (0.00–27.455); 0.415
Adjuvant treatment (yes, no)	2.5 (0.8–7.6); 0.098	
Recurrence (yes, no)	31.043 (4.06–238.56); 0.001	14.145 (1.431–139.836); 0.023

*OS* overall survival, *DOI* depth of invasion, *LVSI* lympho-vascular space invasion, *PNI* perineural invasion, *TFM* pathological tumor-free margin, *NA* not available, *HR* hazard ratio

## Discussion

Vulvar cancers are rare, and prospective studies on prognostic factors are lacking. Most evidence for prognosis is based on retrospective data or collective reviews because of the limited number of women presenting at early stages. Most studies report the association of a single factor with recurrence or survival and have included both early- and advanced-stage cancers. The present study included squamous vulvar cancers (VCs) that underwent upfront surgery and investigated all prognostic factors with probable implications on the outcome. Our study observed that a tumor-free margin is associated with higher recurrence rates, and the need for tailoring surgical management and/or the addition of adjuvant therapy should be evaluated in this subgroup. Depth of stromal invasion and the presence of LVSI are also important risk features to consider when offering a further treatment plan. In our study, these two factors had a significant association with DFS and OS on univariate analysis. However, it should be noted that LVSI positivity was seen in very few patients. We could not establish the consequences of groin node positivity due to the small number of patients with positive status.

Surgery is the cornerstone of the treatment of nonmetastatic vulvar squamous cell cancer. Despite radical treatment, local recurrences are very common in up to 40% of cases [4]. Of these patients, 43–72% will develop a second local recurrence, and subsequently, 57% will have a third or more local recurrences. It has been shown that disease-specific survival decreases from 90 to 69% in patients after a local recurrence. Knowledge of prognostic factors is needed to reduce treatment-related morbidity while improving survival. While stage and lymph node metastases are the most accepted risk factors for recurrence and survival, the importance of TFM, DOI, LVSI, and PNI has yet to be established, as several studies have shown contradictory results [5]. Margin status, tumor grade, and age of the patient were also found to be of considerable predictive significance in a few studies [6].

One of the most debated prognostic factors is the minimal pathologic tumor-free margin distance. Worldwide, a pathologic tumor-free margin of ≥ 8 mm has been advocated as a safe margin. Based on a systematic review of the literature, there seems to be no lower limit (apart from involved margins) below which further treatment (either re-excision or adjuvant radiotherapy) to the vulva should be recommended [5]. A retrospective analysis of 133 patients revealed that the tumor-free margin was not an independent prognostic factor for recurrence or survival, using either an 8-mm cutoff or as a continuous variable [4]. Pathologic tumor-free margin distance did not influence the risk of local recurrence (hazard ratio [HR] 1.03), even using cutoffs of 8, 5, or 3 mm. [7]. Peunis et al., in their retrospective analysis, therefore concluded that a resection margin < 8 mm in vulvar squamous cell carcinoma (SCC) can be accepted, especially in tumors located close to the clitoris, urethra, or anus [8]. The most important factor for local recurrence was the presence of lichen sclerosus [8]. Bedell et al. observed that there was no difference in local recurrence-free survival (RFS) and overall survival (OS) between patients who received re-excision/vulvar radiation for positive or close margin and patients who received no further therapy [9]. In our study, 23 cases (28.4%) had a pathologic tumor-free margin < 10 mm. It was found to have a significant correlation only with increased recurrence (HR 4.0) and not with OS (HR 1.9, p = 0.267). Patients with a close margin < 5 mm were given adjuvant radiotherapy in our study. None of these patients underwent re-excision due to anatomical reasons. Adjuvant therapy for close or positive margins may be a major confounder observed in our study for estimating local recurrence risk.

Several societies have recommended different acceptable tumor-free margins. While the National Comprehensive Cancer Network (NCCN) [10] and the International Federation of Gynecology and Obstetrics (FIGO) [11] recommend a clinical gross margin of 1–2 cm, yielding a histologic margin clearance of > 8 mm (after tissue shrinkage with formalin fixation), the European Society for Gynaecological Oncology (ESGO) suggests a more conservative margin to preserve the function of midline structures (clitoris, urethra, anus) [12]. The German guidelines (Arbeitsgemeinschaft Gynäkologische Onkologie [AGO], Study Group for Gynecologic Oncology) recommend a 3-mm margin clearance [13]. FIGO defines a close margin as < 5 mm and advises re-excision or adjuvant radiotherapy for close margins [11]. Similarly, NCCN recommends re-excision or adjuvant radiotherapy for positive margins, but observation with close regular follow-up may be a reasonable option for close margins in invasive cancer. The definition of a close margin varies between 1 and 8 mm in formalin-fixed tissue in various studies [10].

NCCN recommends inguinofemoral lymphadenectomy for all patients with FIGO stage IB–II disease because of an increased risk of lymph node metastases of more than 8% [10]. The risk of lymph node metastasis increases as the depth of invasion increases. It is considered to be 7–8% for 1.1–3.0 mm invasion and 26–34% for > 3 mm invasion [6]. In early-stage vulvar cancers operated upfront, groin lymph node metastasis is identified in 22.6% of patients [4]. Groin node dissection may be avoided in early stages (≤ 2 cm size with ≤ 1 mm depth of invasion) because the risk of lymph node metastases is negligible in this group. A Swedish study reported worse survival in stage IB–II disease where groin node staging was omitted [14]. In our study, lymph node dissection was not performed in 22.2% of cases. The main reasons were that primary surgery was performed at an outside institution and the patients were referred to our institution for adjuvant therapy (6 cases, 33.3%); lymph node dissection was not indicated (6 cases, 33.3%); or it was safer to omit dissection because of patients’ frailty and comorbidities.

Sentinel lymph node dissection is an alternative method to avoid the high morbidity associated with complete groin node dissection [10]. Morbidity may include infection (21%), lymphocyst (11–40%), or limb lymphedema (14–49%) [15]. Ideal candidates for the sentinel node approach are patients with a unifocal lesion, a tumor size less than 4 cm, and no suspicious groin nodes on clinical or radiologic examination. Dual dye (radiocolloid and blue dye) is preferred as it increases the sensitivity of this method [10]. In recent years, indocyanine green (ICG) has emerged as a new oncologic marker, improving SLN detection in breast, cervical, and endometrial cancers. Studies comparing ICG with radiocolloid are ongoing; however, prospective, randomized, multicenter studies are warranted to assess its safety and accuracy in detecting sentinel lymph nodes in VCs.

The first GROINSS-V study investigated the safety and clinical applicability of the sentinel lymph node procedure in VCs and showed that omitting inguinofemoral lymphadenectomy was safe in early-stage vulvar cancer patients with a negative sentinel lymph node, with an impressive reduction in treatment-related morbidity [16]. GROINSS-V-II further investigated whether radiotherapy could be a safe alternative for inguinofemoral lymphadenectomy in patients with a metastatic sentinel lymph node [16]. This study showed that radiotherapy in patients with sentinel lymph node micrometastases (≤ 2 mm) was safe in terms of groin recurrence rate and had less treatment-related morbidity. GROINSS-V-III recently started including patients. This study investigates the effectiveness and safety of chemoradiation in patients with a macrometastasis (> 2 mm) in the sentinel lymph node [16].

Groin lymph node metastasis is an independent significant prognostic factor for recurrence and survival [4, 5, 17]. It is considered the most important factor for survival [10]. The AGO-CaRE-1 study reported a 3-year progression-free survival (PFS) rate of 35.2% for node-positive patients and an OS rate of 56.2%, compared with 75.2% and 90.2% in node-negative patients [18]. In our study, groin node metastases were identified in 23.5% of cases. The presence of groin node metastases was a significant adverse factor for both DFS (HR 11.5) and OS (HR 7.4).

In a systematic review of clinicopathologic factors for local recurrence after surgery for early-staged vulvar SCC, the prognostic relevance for local recurrence of vulvar carcinoma of all analyzed variables remained equivocal, including a pathologic tumor-free margin distance of 8 mm, grade of differentiation, tumor size, depth of invasion, and lympho-vascular space invasion [5].

In a retrospective analysis of 47 patients who underwent surgical treatment, seven prognostic factors were analyzed in relation to local tumor recurrence: tumor size, margin distance, depth of invasion, lympho-vascular space involvement (LVSI), midline involvement, metastatic lymph nodes, and FIGO stage [19]. All prognostic factors were found to be statistically significant with respect to the risk of local recurrence. The highest risk of local recurrence was observed for a depth of invasion > 5 mm (HR 12.42) and the presence of LVSI (HR 10.83). Depth of stromal invasion has been associated with nodal metastases, with the risk significantly increasing to 26.7% for tumors with stromal invasion > 3 mm [20]. Studies evaluating DOI and its impact on recurrence and survival have used highly variable cutoff values. Depth of stromal invasion is an independent predictor of poor outcome in various studies [20–23]. We observed that DOI was a significant factor for both DFS and OS in our study. Tumor sizes of ≤ 2 cm and > 2 cm were found to be prognostically significant in several studies [20, 24, 25], but we did not find size criteria to be a significant prognostic factor in our study. LVSI as a poor prognostic factor has not yet been proven [2]. Some retrospective analyses have found it to be associated with increased local recurrence [22]. Only five cases (6.2%) in our study had LVSI, so any conclusion relating to recurrence or survival may be erroneous.

Perineural invasion (PNI) is detected in close to 28–30% of vulvar cancers, including both early and advanced stages [26, 27]. In a retrospective analysis by Long et al., PNI was significantly associated with DFS and OS only on univariate analysis [26]. This was explained by the association of PNI with increased size and depth of invasion, LVSI of the primary tumor, advanced stage, and nodal involvement. While other studies have shown PNI to be an independent prognostic factor for recurrence and survival [27], it is advised to report PNI in all histopathologic reports and also consider it when planning adjuvant treatment [27, 28]. In our study, PNI was present in 6.2% (5 cases), and no information regarding PNI was available in 19.8% of cases. It was found to be significantly associated with DFS but not significantly associated with OS.

Adjuvant treatment for vulvar cancers is based on primary tumor risk factors and nodal metastases. Risk factors that may warrant adjuvant therapy to the primary site include close or positive margins, LVSI, tumor size, depth of invasion, and the pattern of invasion (spray/diffuse) [10]. These criteria are not universally agreed upon, and hence adjuvant radiation to the primary site in the absence of nodal metastases is usually individualized based on institutional protocol. A recent Surveillance, Epidemiology, and End Results (SEER)–Medicare-linked data analysis examined outcomes of node-positive vulvar cancer patients who received adjuvant radiation and found that, compared to surgery alone, adjuvant radiation improved survival [29]. The benefit of adjuvant radiotherapy is higher in the presence of more than two positive nodes [10]. An analysis of data from 1797 patients with node-positive cancer from the National Cancer Data Base (NCDB) database showed improved survival with the addition of chemotherapy to adjuvant radiotherapy versus radiotherapy alone (44 months vs. 29.7 months) [30]. In our study, the main indication for adjuvant treatment was lymph node metastases (16/19 cases, 84.2%). These patients received external beam radiotherapy (EBRT) to the pelvis, groin, and vulva. The addition of chemotherapy was based on age, glomerular filtration rate (GFR), and the presence of comorbidities. Only radiation was given in 13 cases (68.4%), and 5 cases (26.3%) received chemoradiotherapy (CTRT). Two patients received radiation to the vulva because of close margins, and one stage IB patient received EBRT to the pelvis, groin, and primary site because of another synchronous lesion in the vagina. Adjuvant therapy was delayed for eight weeks in one patient because of delayed wound healing. She received only adjuvant chemotherapy because of progressive disease.

The overall recurrence rate in our study was 33.3%, and the median time to recurrence was 36.14 months. Recurrence sites were local (13 cases, 48.1%), groin recurrence with or without local disease in 9 cases (33.3%), and 5 cases (18.5%) developed distant recurrence. Treatment for recurrence consisted of palliative chemotherapy or symptomatic therapy in 14 cases (51.8%), surgery with or without adjuvant radiation in 7 cases (25.9%), and radiotherapy plus or minus chemotherapy without prior surgery in 6 cases (22.2%). Patients who experienced recurrence had poorer survival compared to those without recurrence. A Swedish nationwide population-based study reported that the 2-year OS post-recurrence was 57.8% for local recurrence, 17.2% for groin recurrence, and 0% for distant recurrences [14]. The long-term follow-up of GROINSS-V also showed that the 10-year disease-specific survival rates in cases of local recurrence were reduced from 90.4 to 68.7% [31].

This study is limited by its retrospective, single-center design. However, the present results enable the identification of patients at higher risk of recurrence for whom more aggressive treatment or surveillance may be warranted. Further treatment attempts and constant attention are needed to improve outcomes for VC patients.

## Conclusion

Vulvar cancers are rare, and surgical excision is the primary treatment of choice. This study emphasizes the various prognostic factors impacting survival and recurrence in vulvar cancers. Local tumor factors such as TFM, DOI, and LVSI, along with lymph node status, determine oncological outcomes in vulvar cancer. However, there is a paucity of multicenter data on vulvar cancers due to the rarity of the disease. Collaborative reporting of similar-stage VC patients is needed to establish undeniable evidence.
